# The Role of Microorganisms in the Etiopathogenesis of Demyelinating Diseases

**DOI:** 10.3390/life13061309

**Published:** 2023-06-01

**Authors:** Jessica Frau, Giancarlo Coghe, Lorena Lorefice, Giuseppe Fenu, Eleonora Cocco

**Affiliations:** 1 Multiple Sclerosis Centre, ASL Cagliari, 09126 Cagliari, Italy; gccoghe@gmail.com (G.C.); lorena.lorefice@hotmail.it (L.L.); ecocco@unica.it (E.C.); 2 Neurologia, ARNAS G. Brotzu, 09047 Selargius, Italy; giusefenu@gmail.com; 3 Department of Medical Sciences and Public Health, University of Cagliari, 09124 Cagliari, Italy

**Keywords:** multiple sclerosis, neuromyelitis optica, myelin oligodendrocyte glycoprotein-associated diseases, infections, viruses, Epstein–Barr virus, human endogenous retrovirus

## Abstract

Multiple sclerosis (MS), neuromyelitis optica (NMO) and myelin oligodendrocyte glycoprotein antibody disease (MOGAD) are inflammatory diseases of the central nervous system (CNS) with a multifactorial aetiology. Environmental factors are important for their development and microorganisms could play a determining role. They can directly damage the CNS, but their interaction with the immune system is even more important. The possible mechanisms involved include molecular mimicry, epitope spreading, bystander activation and the dual cell receptor theory. The role of Epstein–Barr virus (EBV) in MS has been definitely established, since being seropositive is a necessary condition for the onset of MS. EBV interacts with genetic and environmental factors, such as low levels of vitamin D and human endogenous retrovirus (HERV), another microorganism implicated in the disease. Many cases of onset or exacerbation of neuromyelitis optica spectrum disorder (NMOSD) have been described after infection with Mycobacterium tuberculosis, EBV and human immunodeficiency virus; however, no definite association with a virus has been found. A possible role has been suggested for Helicobacter pylori, in particular in individuals with aquaporin 4 antibodies. The onset of MOGAD could occur after an infection, mainly in the monophasic course of the disease. A role for the HERV in MOGAD has been hypothesized. In this review, we examined the current understanding of the involvement of infectious factors in MS, NMO and MOGAD. Our objective was to elucidate the roles of each microorganism in initiating the diseases and influencing their clinical progression. We aimed to discuss both the infectious factors that have a well-established role and those that have yielded conflicting results across various studies.

## 1. Introduction

Demyelinating diseases of the central nervous system (CNS) are a group of overlapping syndromes characterized by immune-mediated inflammation of the brain and spinal cord. They mainly affect young adult people and their frequency varies worldwide, with multiple sclerosis (MS), neuromyelitis optica spectrum disorders (NMOSD) and myelin oligodendrocyte glycoprotein antibody disease (MOGAD) being the most important [1].

The cause of demyelinating diseases is unknown, but an interaction between genetics and the environment has been established, especially for MS. Among the environmental factors, the main risk factors for MS are vitamin D deficiency, obesity, smoking and infections [2].

Knowledge of the importance of infectious agents in the triggering of autoimmunity is very old, with MS having been postulated to be triggered by a microorganism as early as 1900 [3].

The role of persistent slow infections in MS has been suspected since 1975 when elevated titers of antibodies against rubeola, vaccinia and measles viruses were found in the blood and cerebrospinal fluid (CSF) of patients with MS, subacute sclerosing panencephalitis and NMOSD [4].

In some autoimmune diseases such as MS and type 1 diabetes, infections during pregnancy have also been observed to play a role. In fact, patients with MS exhibit a month of birth seasonality that is different from that of healthy controls, suggesting that perinatal infections could trigger autoimmunity [5,6]. Two mechanisms have been postulated to explain the involvement of the fetus during the viral infection affecting the mother. In the first one, the inflammatory immune response caused by the viral infection in the mother is followed by a transmission of cytokines to the fetus. Alternatively, the viruses could be transmitted directly from the mother to the fetus [7]. On the contrary, there is a possibility of other environmental factors playing a role in the observed impact of the birth season. Specifically, studies have revealed that in the northern hemisphere, this impact correlates with latitude- and climate-related variables such as sunshine duration. Additionally, inadequate levels of vitamin D in the mother may increase the risk of MS in the infant and these levels are closely linked to sun exposure, which varies seasonally [8].

Viruses can damage the infected organism and trigger autoimmunity in different pathways [9].

The most plausible mechanism that could explain the role of infections in the triggering of autoimmunity is molecular mimicry, which consists of a cross-reaction between self and nonself epitopes, leading to their presentation by antigen-presenting cells to autoreactive CD4+ T cells [10,11].

**Figure 1:**
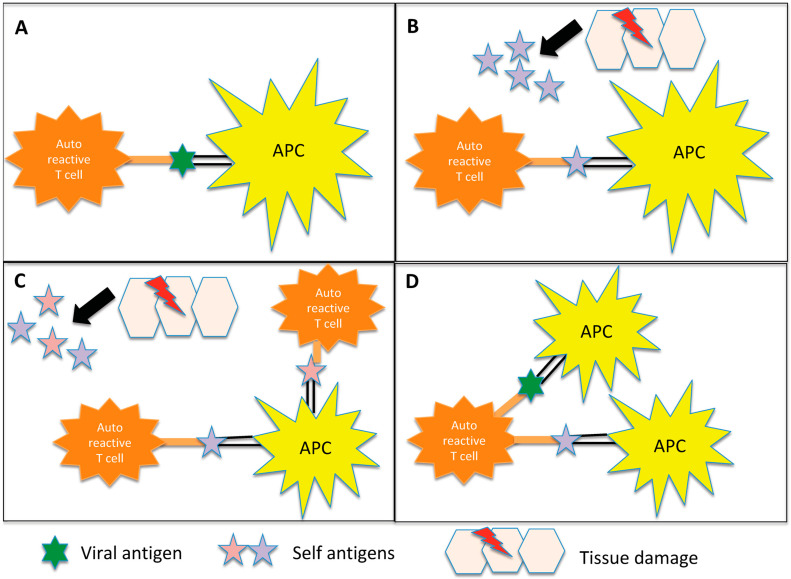
Representation of the potential inflammation-mediated mechanisms underlying autoimmunity triggered by infections. (**A**) Molecular mimicry: In certain instances, the antigens found on pathogens may bear a resemblance to self-antigens present in the body. This similarity can result in the activation of autoreactive T cells, which erroneously identify self-antigens as foreign. Notably, in demyelinating diseases, antigen-presenting cells (APCs) present myelin components to autoreactive CD4+ T cells, initiating the cascade of autoimmune responses. (**B**) Bystander activation: The virus induces a profound inflammatory response, resulting in significant damage to the surrounding tissue. Consequently, additional autoantigens become exposed and antigen-presenting cells (APCs) present these autoantigens to autoreactive CD4+ T cells. (**C**) Epitope spreading: Initially, the immune response may target a specific antigen derived from the infecting pathogen. However, as time progresses, the immune response can expand to encompass other self-antigens that share structural similarities or associations with the initial target antigen. In demyelinating diseases, for instance, viral infections can lead to the destruction of oligodendrocytes. Subsequent fragmentation of myelin in the inflammatory milieu exposes additional antigens, contributing to a self-perpetuating cycle of myelin destruction. (**D**) Dual T cell receptor: Certain T lymphocytes possess the capability to express multiple T cell receptors, allowing them to recognize both viral and myelin antigens. Consequently, these dual-specificity T cells can activate responses against both types of antigens simultaneously.

Other pathways include direct neural toxicity, which is not mediated by inflammation [9]; bystander activation, which is an abnormal immune activation after tissue damage involving the exposure of normally hidden autoantigens and the production of other autoreactive T cells [10]; and epitope spreading, which consists of the release of myelin fragments after their disruption in the inflammatory environment, resulting in the exposure of additional epitopes [12,13]. Another mechanism is known as “dual T-cell receptors” and involves T cells that carry two different receptors with specificity for myelin and for microorganism epitopes, respectively [13] (Figure 1).

Even if viruses are considered as the microorganisms that are more implicated in the pathogenesis of demyelinating diseases, it has recently been suggested that fungi also play a role. Considering that CD4+ T cells are indispensable to protecting the organism against fungal infections, an overactivation of these immune cells could potentially lead to autoimmunity [14].

Many factors related to infection have been implicated in demyelinating diseases, some of which have only been described in a few populations [9,15,16,17].

In this review, our aim was to comprehensively discuss the role of infectious factors in MS, NMOSD and MOGAD individually. We conducted thorough research on each microorganism, presenting the current knowledge and hypotheses regarding their association with the onset and clinical course of these three diseases. We emphasized instances where the role of infections is well-established, backed by robust studies, as well as situations where different studies have yielded conflicting results. Additionally, we explored the potential involvement of disease-modifying treatments (DMTs) in managing the infection, particularly focusing on infectious factors strongly associated with MS.

## 2. MS

MS is the most common demyelinating disease of the CNS and its pathogenesis is multifactorial. Indeed, although a genetic predisposition is required, this in itself is not a sufficient condition for its occurrence. Many environmental factors, particularly acting during the first decades of life, are determinants of disease development, among which microorganisms play an important role [18,19]. MS is therefore a demyelinating disease for which strong evidence of the role of infections in its aetiopathogenesis is available. Several viruses, including rabies, coronavirus, measles, torque teno virus, herpes viruses and parainfluenza have been hypothesized to be important in MS [20].

**Figure 2:**
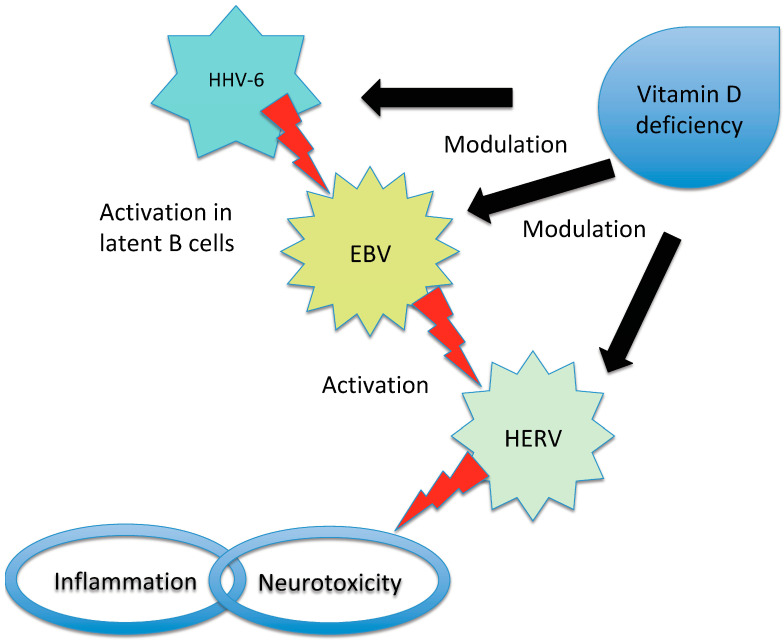
Representation of the interaction between EBV, HHV-6, HERV and vitamin D in triggering MS. Vitamin D deficiency has been proposed to influence the risk of MS in individuals infected with EBV due to the overlap between the receptor-binding sites of EBNA-2 (a protein produced by EBV) and vitamin D. Additionally, high levels of vitamin D may inhibit EBV infection through apoptosis, while HHV-6 can activate latent EBV in B cells. In patients with MS, an interaction between vitamin D and HERV has also been observed. There is an inverse correlation between HERV-W DNA levels and vitamin D levels and high vitamin D levels may inhibit HERV transactivation. Furthermore, low vitamin D levels increase the risk of MS in individuals with high titers of anti-HHV-6A antibodies. EBV has the ability to induce the expression of certain HERV genes and the activation of HERV-W during infectious mononucleosis has been proposed as a potential effector in the pathogenesis of MS. It is important to note that while these relationships have been identified, the precise mechanisms and their significance in MS development and progression require further investigation. The interplay between EBV, HHV, HERV and vitamin D in the context of MS is complex and multifaceted.

The microorganism with the strongest association with MS is the Epstein–Barr virus (EBV) [21,22], but it is possible that more than one agent may be involved [23]. Moreover, viruses can act as triggers or cofactors and strict interactions have been described among several microorganisms and between them and other environmental and genetic factors, such as among EBV, vitamin D receptor and HERV expression [9,24] (Figure 2).

Table 1 shows a selection of studies investigating the role of infectious factors in MS.

### 2.1. EBV

The presence of higher titers of antibodies against EBV in patients with MS compared to healthy controls has been understood since the eighties [25,26,27].

This virus is present in almost all patients with MS [28] and the risk of developing the disease is significantly higher if the patient has experienced a previous mononucleosis infection [91].

Recently, the role of EBV in the disease has been definitely established by a large cohort study involving more than 10 million military personnel who have been monitored for years [21]. The authors found EBV seropositivity in all but one of the 801 cases of MS diagnosed during follow-up and the risk of developing the disease for the infected persons was 32-fold higher compared to that for seronegative cases. Other viruses were not implicated in MS in this study. Interestingly, a cause-and-effect relationship between EBV and MS has been demonstrated by the presence in MS patients of high levels of neurofilament light chains, a serum biomarker of neurodegeneration, only after but never before EBV infection. This study definitively established that individuals that are seronegative for EBV have a low risk of developing MS [21]. In EBV-infected individuals, however, antibody titers against EBV are strong prognostic markers of MS onset [29].

Cross-reactivity with myelin basic protein has been detected in the serum of patients with antibodies against epitopes from EBV proteins such as Epstein–Barr nuclear antigen (EBNA)-1 and DNA polymerase [30,31].

Despite the detection of EBV-specific oligoclonal bands (OCBs) in the CSF and the finding that OCBs from patients with MS bind to specific EBV proteins [32,33,34], the actual presence of the virus in the CNS of these patients has not been well established and the studies have yielded contradicting results [92].

The onset of MS may occur decades after the primary EBV infection [35,36]. This timeframe reflects the prodromal phase of MS [93] and it is possible that, during this period, EBV may interact with the immune system of the host through affinity maturation and clonal expansion of B cells, expansion of EBV-infected B cell reservoirs and epitope spreading, thus contributing to MS development [21].

It has been hypothesized that EBV could act through molecular mimicry, with B cells losing the episomic EBV DNA after replication but retaining the “forbidden” epitope recognition [94]. According to another theory, EBV could disrupt the blood–brain barrier during primary infection, allowing autoimmune cells to enter the CNS and leading to local inflammation [95]. The possibility of a persistent EBV infection in the CNS with activation of the immune response and subsequent CNS tissue damage is less probable [37,96]. However, B cells activated by EBV are preferentially recruited to the CNS and submucosal secondary lymphoid organs, thus stimulating autoimmune T cells, either in the CNS or in intestinal lymphoid organs [97]. After the initial damage in the CNS, autoantibodies produced by the infected B cells could target the myelin sheath and cause the release of myelin fragments, which in turn could stimulate the T cells to continue the degradation process [98].

Moreover, EBV could activate the human endogenous retrovirus (HERV), which has pro-inflammatory and neurotoxic properties [99].

The genetic factors involved in EBV control are becoming increasingly important. The main hypothesis regarding the role of EBV as a “key pathogenic event” in MS involves an altered balance between the virus and the immune system of the host in genetically predisposed individuals [100]. Indeed, the control of EBV within the human body is influenced by a combination of genetic and environmental factors. These factors, to some extent, are associated with age and sex and can influence the immune response against EBV [100].

It has been suggested that mutations in EBNA-2 could influence the host response and the risk of developing MS [38]. Some major histocompatibility complex (MHC) class II alleles predisposing to MS function as coreceptors for EBV entry into B cells [39], but other MS loci are also involved in the response to EBNA-2 [100] and there is an interaction between EBNA-1 antibody titers and HLA-DRB1*1501 [101]. It has been calculated that the risk of MS is more than 20-fold higher if HLA-DRB1*1501 is present in combination with high titers of EBNA-1 antibodies [40]. Furthermore, recent studies have observed that EBV, through the utilization of specific virulence factors, may possess the ability to manipulate MS susceptibility genes via epigenetic regulation [102].

Another piece of evidence is the presence of a complex interaction between EBV and environmental factors associated with MS, such as low vitamin D levels, obesity in childhood and adolescence and smoking. Their role in the modulation of MS risk after EBV infection has been hypothesized [21]. For example, there is an overlap between the receptor-binding sites of EBNA-2 and vitamin D, which suggests that the role of EBV in the increase in MS risk could be modulated by vitamin D deficiency. Furthermore, vitamin D could inhibit EBV infection via apoptosis and EBV-EBNA-3 could hamper the function of the vitamin D receptor [41].

Although the importance of EBV in the induction of MS is clear, there is no agreement about its role in the course of the disease or in the conversion from clinically isolated syndrome (CIS) to MS [103]. Some studies have agreed on the correlation between EBNA-1 antibody titers and radiological disease activity [42,43].

Nevertheless, there is evidence of DMTs, such as interferon beta, teriflunomide and ocrelizumab, playing a role in decreasing the immune response to EBV or inhibiting its replication in “in vitro” studies [44,45]. Therefore, the effectiveness of DMTs could be partly attributed to their capacity to modify the interaction between EBV and the immune system of the host.

In addition, new drugs such as Bruton tyrosine kinase inhibitors suppress the activation of EBV lytic infection [104], while others still being developed represent EBV-specific T cell immunotherapy aiming to reinforce the immune control of this virus that is deficient in patients with MS [100].

Hence, as our understanding of the role of EBV in terms of its interaction with the immune system and its impact on the clinical course continues to expand, clinical studies focusing on targeting EBV are currently underway [105].

A phase I trial has commenced, employing autologous EBV-specific T cell therapy, in patients with progressive MS and CIS, demonstrating promising results in terms of safety and efficacy in reducing disability. Additionally, studies on the noncyclic nucleoside analogue tenofovir alafenamide have revealed its ability to inhibit EBV DNA polymerase. Based on these findings, a clinical trial is currently underway to assess the use of this drug as an add-on to ocrelizumab, evaluating its efficacy in alleviating MS symptoms and promoting neuroprotection [102].

Owing to the primary role of EBV in the pathogenesis of MS, new prophylactic vaccines against the virus are being tested in healthy young adults [106]. The main aim of the vaccines should be to prevent EBV infection, but a vaccine that controls the immune response to EBV could also reduce the frequency of MS. On the other hand, research on vaccines is complicated due to potential adverse events such as an aberrant immune response that could trigger autoimmunity or transient protection against EBV that could only delay the infection and the onset of MS [21].

### 2.2. HERV

HERV are a class of retroviruses incorporated in the human genome millions of years ago and they represent up to 8% of the human genome. HERV are classified into various classes and families, with each family being designated by appending a letter after the acronym “HERV”. The letter corresponds to the transfer RNA specificity of the primer-binding site. The activation of the K, H and W types by a variety of different stimuli (i.e., infection with EBV, human herpes virus-6 and other viruses) could trigger demyelination in MS [107,108,109]. It is estimated that 8% of the entire human genome is constituted by some types of HERV, which normally have a regulatory function for human gene expression [110]. Nevertheless, they are abnormally expressed in some types of cancers and autoimmune diseases [20].

HERV-W is the type most closely implicated in MS pathogenesis and its envelope protein has been detected in the serum, brain, perivascular infiltrates and macrophages of patients with MS [46,47,48]. Moreover, HERV mRNA has been found in the brain lesions, CSF and blood cells of individuals with MS [49,50]. It has been demonstrated that the HERV-W envelope can stimulate microglia to damage myelinated axons, activate monocytes and endothelial cells and drive the production of pro-inflammatory cytokines responsible for demyelination and neurodegeneration [24,48].

HERV-W envelope epitopes are expressed on activated B cells and monocytes in individuals with MS, suggesting cross-reactivity through molecular mimicry [51,53]. The HERV-W envelope protein can be colocalized with oligodendrocyte progenitors in normal white matter, resulting in disrupted myelin repair, abnormal demyelination and development of MS [54].

Other HERV types, such as HERV-K and -H, have been reported to be associated with MS. In particular, the levels of some HERV-K gag genes are increased in mononuclear cells and in the brains of individuals with MS [52,111]. The HERV-H envelope and gag proteins have been reported to be present in the serum of MS patients [55] and when patients with active disease were compared to those with inactive MS and with healthy controls, both the HERV-H envelope protein and an HERV-H single nucleotide polymorphisms were found only in immune blood cells derived from the first group [51,52].

HERV-W appears to be implicated not only in the pathogenesis of MS, but also in its clinical course. Indeed, patients with a high level of disability or those in phases of high disease activity have more active loci for the HERV-W envelope compared to other patients with MS and to healthy controls [20].

It has been hypothesized that HERV-W is activated by EBV during infectious mononucleosis, thereby acting as an effector in MS pathogenesis [56]. EBV appears to induce the expression of some HERV genes in both patients with MS and in healthy controls [50,57]. HERV may activate the host immune response by acting as a superantigen or a toll-like receptor agonist [54,112].

In addition, other viruses such as varicella-zoster (VZV), herpes simplex-1 and herpes virus-6 could activate HERV [24].

An inverse correlation between HERV-W DNA and vitamin D levels has been reported in individuals with MS, suggesting a potential interaction between HERV and vitamin D [58]. Moreover, high levels of vitamin D could inhibit HERV transactivation [113]. Thus, both EBV and vitamin D levels could modify HERV expression and HERV could be considered the missing link between these factors and the induction of inflammation that takes place in MS [24].

Interestingly, as a further suggestion regarding the role of HERV in MS, DMTs such as interferon beta, natalizumab, rituximab and fingolimod may decrease the expression of HERV-W [59,60,114]. Additionally, a new drug tested in a phase II clinical trial for MS, known as GNbCA1, has been shown to block the HERV-W-dependent inflammatory cascade [61,62].

### 2.3. Human Herpes Virus (HHV)

Higher titers of antibodies against herpes simplex virus-2 have been reported in patients with MS, whereas herpes simplex virus-1 has been reported in pediatric cases and in CIS, but not in adult MS [115].

The HHV subtype with stronger evidence of implication in MS is HHV-6. HHV-6 infects the majority of the population before one year of age and comprises two species: HHV-6A and HHV-6B [116]. The detection of HHV-6 DNA in MS lesions, CSF and blood cells, the observation of high antibody titers in patients with MS and the identification of OCB specificity against HHV-6 have suggested an association between infection with this microorganism and the disease [63,64].

Although other studies have reported no association between HHV-6 and MS risk [117], a clear difference between patients with MS and healthy controls has been established after a thorough metanalysis in terms of anti-HHV-6 antibody titers and HHV-6 mRNA and DNA [118]. Furthermore, the antibody titer was associated with an enhanced risk of relapse [65].

Among the two subtypes, HHV-6A is more neurotrophic and it has been observed that the increased cellular immune response and increased HHV-6 DNA detected in the serum and CSF of patients with MS corresponds to subtype A and not B [66,67].

Increased anti-HHV-6 IgM has been reported in the early stages of the disease, suggesting an important role for the virus during this phase [119].

HHV-6 stimulates the microglia to produce pro-inflammatory molecules and activates latent EBV in B cells [9,120]. However, it is unclear how the virus induces these autoimmune processes. One of the hypotheses involves molecular mimicry through cross-reactivity with basic myelin proteins [68]. Other pathogenic pathways include incorporation into the host cell leading to the expression of host proteins in the virus envelope, direct infection of the CNS with primary local injury and triggering of the inflammation cascade [119,121].

An interaction with genetic factors has also been reported for HHV-6. In particular, the presence of HLA DRB1*1501 in combination with high anti-HHV-6 antibody titers and with the absence of the protective allele HLA-A*0201 increases the risk of MS [69]. Moreover, environmental risk factors for MS, such as low vitamin D levels, low UV irradiation, smoking and EBV infection, increase the risk of MS in individuals with high titers of anti-HHV-6A antibodies [70,71]. In contrast, there is an inverse interaction with cytomegalovirus (CMV) infection: individuals with low levels of anti-CMV antibodies and high titers of anti-EBV and anti-HHV-6A antibodies have a 15-fold higher risk of developing MS compared to those with high anti-CMV and low anti-EBV and anti-HHV-6A antibody levels [89].

### 2.4. Gut Microbiota

There is clear evidence of a link between the gut microbiota and MS, with both bacteria and fungi playing an important role [122,123]. Different microorganisms comprise the gut microbiota, with *Firmicutes, Bacteroidetes, Proteobacteria* and *Actinobacteria* representing more than 97% of it [124]. Its composition is influenced by several internal and external factors, including diet, lifestyle and physical activity [125]. Disruption in the normal composition of gut microbiota, known as “dysbiosis”, has been related to many diseases [126]. The complex interplay between the CNS and the gut has led to the concept of the “gut-brain axis” and it has been noted that the development of the immune system from birth depends on the composition of the gut microbiota [127]. The gut microbiota of individuals with MS has some peculiarities compared to that of healthy individuals, such as an increased abundance of *Akkermansia, Ruminococcus, Blautia* and *Bifidobacterium*, while other microorganisms such as *Bacteroides, Parabacteroides, Prevotella* and *Lactobacillus* are less represented [128]. The first group regulates the immune response mediated by T cells, promoting a pro-inflammatory and autoimmune environment both in vivo and in vitro. In contrast, the bacterial species that are less abundant in the gut of patients with MS have anti-inflammatory properties [72].

There is evidence of the amelioration of MS symptoms through the manipulation of the gut microbiota composition with probiotic supplementation, the use of antibiotics or changes in dietary habits. Moreover, some pilot studies have confirmed the promising role of microbiota transplantation [129].

It has recently been suggested that both DMTs and vitamin D may have an impact on the gut microbiota [130]. Indeed, dimethylfumarate can promote an increase in *Bacteroides* and a decrease in two clostridial families, whereas interferon beta can cause an increase in *Prevotella* [73,74].

Other studies have explored the effect of high-efficacy DMTs such as natalizumab, anti-CD20 antibodies and fingolimod and changes in the gut microbiota composition have been found in patients that were treated with these drugs [75]. However, it is not clear whether these changes in the composition of the microbiota are associated with altered function, or whether they could affect the efficacy of DMTs [131].

### 2.5. Fungi

Recent studies have shown that fungi may play an important role in the pathogenesis of MS [14] and the finding that genes involved in innate immunity are associated with the disease suggests a link between MS and dysregulation of the innate immune system [18].

*Candida albicans* is the fungus most closely associated with MS according to the available evidence. *Candida* infection is more frequent in MS patients compared to healthy individuals and correlates with disease severity. Antibodies against *Candida* have also been found in the CSF of individuals with MS [77,78,79]. This association may be the result of molecular mimicry, with memory B cells recognizing both *Candida* and CNS epitopes [17]. It has been hypothesized that the higher prevalence of MS among women could be partially explained by the higher titers of antibodies against *Candida* present in healthy women compared to men [132].

Another fungus isolated from the majority of individuals with MS is *Trichosporon mucoid*, while both *Aspergillus* and *Saccharomyces* have been shown to be increased in the gut microbiota of MS patients compared to healthy controls [76,77].

Fungal toxins could have a direct role in the development of the disease by damaging oligodendrocytes and astrocytes, but the peripheral action of fungi on the immune system may also make a contribution [14,133].

Notably, T helper 17 cells play a crucial role in MS pathogenesis and they are also important for antifungal immunity [134,135]. *Candida albicans* mannoproteins promote the production of interleukin 17 (IL-17) and even a low fungal load inside the CNS can trigger high levels of a T helper-17-mediated immune response, thus provoking an important increase in IL-17 [136,137]. Another finding suggesting the role of fungi inside the CNS in the development of MS is the presence of high levels of the antimicrobial protein calproctectin in the CSF during relapses [80]. The effectiveness of antifungal therapy in MS remains unclear, but in a study from more than 40 years ago, some improvement in MS symptoms was observed in a few patients after treatment with antifungal drugs [81]. Currently, one of the drugs used in clinical practice to treat MS (dimethylfumarate) is also a fungicide [17].

### 2.6. Mycobacterium avium Subspecies Paratuberculosis (MAP)

To date, this microorganism has been associated with MS only in Sardinia and Japan [82,84]. In a large cohort of Sardinian MS patients, a significantly higher frequency of anti-MAP antibodies and MAP DNA was observed compared to healthy controls [82,83]. Moreover, specific MAP antigens that are homologous with MS-related proteins have been described, suggesting that molecular mimicry and the ability to enhance the immune response are the main pathogenic mechanisms linking MAP to MS development [31,85,86,138].

After the study on the Sardinian cohort, a higher response against MAP was observed in a cohort of Japanese MS patients compared to healthy controls [84]. The prevalence of anti-MAP antibodies in the Sardinian and Japanese cohorts was quite similar [16]. Moreover, the two cohorts had a rare genetic factor in common (HLA-DRB1 04*05), which predisposed them to develop MS symptoms [84].

MAP infection can enhance the risk of MS if other factors, such as genetic predisposition, are present [15]. Nevertheless, no specific association between the predisposing haplotypes and MAP positivity was found in the Sardinian population. On the other hand, lower titers of anti-MAP antibodies have been detected in patients carrying at least one protective haplotype [87].

It has been suggested that the beneficial effects of the Bacillus Calmette–Guerin (BCG) vaccine in MS patients are derived from MAP mitigation [139]. On the other hand, the vaccine should prevent neuroinflammation, contrasting the low exposure to microbes and other pro-inflammatory changes in lifestyle that have occurred in recent decades [140].

This vaccine has been used since 1950 to prevent allergic encephalomyelitis [140]. It has also been tested in MS and CIS patients and it was shown to decrease disease activity assessed by magnetic resonance imaging, as well as to lower the risk of conversion from CIS to clinically defined MS [141,142,143]. More recently, this vaccine has also been proposed as a therapeutic agent against radiologically isolated syndrome and a phase II study is ongoing.

### 2.7. CMV

The active role of CMV in the pathogenesis of MS has been hypothesized [88] and it has been clearly established that it has a protective effect against the disease [22,26,65,90]. This may be related to the capacity of CMV to enhance the immune control of EBV infection, thus reducing the risk of MS development [144].

## 3. NMOSD

NMOSD designates a group of demyelinating syndromes characterized by specific clinical and radiological findings in addition to the presence of anti-aquaporin-4 (AQP4) antibodies in 80–90% of the patients [145]. The prevalence of the disease is increasing, probably because of changes in environmental factors such as the increased use of antibiotics and improvements in hygiene conditions [146].

NMO pathogenesis involves both genetic and environmental factors; however, clear evidence is lacking at the moment. It is likely that factors in the environment trigger the onset of the disease in genetically predisposed individuals [147].

The evidence regarding the role of infections in NMO is not as strong as that available for MS due to the dearth of experimental data, which in turn may be the result of the low prevalence of the disease and the small sample size of the studies. Nevertheless, several infectious agents have been described as potential triggers for disease onset or exacerbation, including CMV, VZV, EBV, hepatitis A virus, human immunodeficiency virus (HIV), *Mycobacterium tuberculosis*, *Mycoplasma pneumoniae*, human T-lymphotropic virus type 1 and dengue virus [148,149]. It has been reported in several cases that the onset of NMO occurs after a viral infection, but the frequency of previous infection is highly variable among different studies, ranging from 15% to 50% [149,150,151].

An analysis of cases of NMOSD reported after infection between 1975 and 2009 showed a high frequency of monophasic course and poor prognosis compared to cases without previous infection [152]. Koga et al. reported a short disease history and prevalence of acute myelitis without optic neuritis in patients with a previous acute infection [149].

It is possible that viral infection may be able to increase blood–brain barrier permeability, thus allowing the entry of autoantibodies into the CNS [149]. Nevertheless, as previously described in the case of MS, viruses trigger autoimmunity through molecular mimicry, bystander activation and/or antigen spreading [153].

The “hygiene hypothesis” has been advanced in support of the role of microorganisms in the pathogenesis of NMOSD. Indeed, daycare attendance has been associated with a low risk of NMOSD, whereas caesarean delivery doubles the risk compared to natural birth [154].

In Table 2, a selection of studies investigating the role of infectious factors in NMOSD is shown.

### 3.1. Tuberculosis (TB)

Many cases of NMO have been reported after or during active TB, in particular with pulmonary involvement [155,158,160]. Notably, Rafai et al. reported on two patients with NMOSD, each presenting distinct clinical observations. In one case, the diagnosis of pulmonary TB closely preceded the onset of NMOSD, whereas in the other patient, neurological symptoms of NMOSD were observed prior to the detection of pulmonary TB positivity in the sputum [158]. An additional remarkable case involved a patient who consistently experienced NMOSD relapses following episodes of active pulmonary TB [155]. Furthermore, among a cohort of Brazilian patients, a history of previous pulmonary TB was identified in 5 out of 24 individuals diagnosed with NMOSD [160].

In a case-control study, a higher frequency of pulmonary TB was observed in patients with NMOSD compared to healthy controls and a close temporal association (with a median of four weeks) was observed between the two diagnoses [157]. However, this finding was not confirmed in a different population [156].

Supporting the role of TB in NMO is the observation of an improvement in neurological symptoms after TB treatment in a cohort of patients with NMOSD who were unresponsive to steroids [159].

### 3.2. Helicobacter pylori (H. pylori)

Contradictory results have been reported in studies regarding the prevalence of this microorganism in patients with NMO.

When AQP4-positive and AQP4-negative NMOSD patients were compared, higher titers of antibodies against *H. pylori* were observed only in the former group [161]. In contrast, another study evaluating patients with NMO found high levels of antibodies against *H. pylori* in both AQP4-positive and AQP4-negative patients when compared to patients with MS and to healthy controls, although the stronger association was with the AQP4-positive group [162]. In another cohort, a higher prevalence of *H. pylori* infection was described in AQP4-positive compared to AQP4-negative patients [163]. Finally, a comparison between AQP4-negative NMOSD patients and healthy controls did not reveal any difference in terms of the frequency of *H. pylori* infection [164]. Considered together, these findings suggest a probable role for *H. pylori* in AQP4-positive patients and a weak association in the case of AQP4-negative patients.

It is possible that the involvement of this bacterium in NMO triggering may not only be through the promotion of cell differentiation in T helper 1 and 17 cells but also through increasing the levels of cytokines and leukocytes and by stimulating mast cells to produce pro-inflammatory cytokines [174]. In contrast to the case of other microorganisms, molecular mimicry between *H. pylori* and self-antigens has not yet been demonstrated [147].

### 3.3. EBV

Data regarding the active role of EBV in NMOSD are not as robust as those in MS. However, the serum and CSF titers of anti-early antigen (EA) IgG antibodies are higher in patients with NMOSD than in those with MS or in healthy controls and they are positively associated with AQP4 levels [165]. The same study showed that anti-EA IgM levels were higher in NMO and MS patients compared to healthy controls, whereas antiviral capsid antigen and anti-EBNA IgG levels were higher in MS patients than in NMO patients or healthy individuals. It has been suggested that pro-inflammatory molecules produced early during EBV infection may exacerbate NMO. The high levels of anti-EA antibodies could indicate active viral replication in patients with NMO and the presence of these antibodies in the CSF is indicative of their local production [163]. When antibodies against other viruses, such as VZV, CMV and HSV, were measured in the serum of the same cohort, no difference between patients with MS and NMO was found [165].

Another study reported that NMO could be associated with the reactivation of EBV and not with a single episode of past infection [166].

### 3.4. HERV

Despite the limited number of studies, there is a general agreement regarding the negative association between antibodies against the HERV-W envelope and NMOSD, particularly in patients that are seropositive for AQP4 IgG. In fact, patients with NMOSD have lower levels of antibodies compared to patients with MS and to healthy individuals [167,168]. The different trends in the levels of these antibodies among patients with MS and NMOSD suggest that this could be used as a potential biomarker to improve discrimination between these two diseases [167].

### 3.5. Gut Microbiota

The roles of *Clostridium perfringens* and *Escherichia coli* in NMOSD have been suggested by their effect in T helper 17 and T regulatory cell balance [147]. Moreover, the presence of *Clostridium perfringens* has been found to be enhanced in individuals with NMO compared to that in patients with MS and healthy controls [169] and potential molecular mimicry between this microorganism and AQP4 has been suggested [170].

### 3.6. Fungi

There is no clear evidence supporting the role of fungi in NMO; however, *Candida albicans* has been reported to promote lymphocyte proliferation in these patients, suggesting a possible exacerbation of NMO after fungal infection [148].

### 3.7. Human Immunodeficiency Virus (HIV)

A separate discussion needs to be conducted regarding HIV infection associated with NMOSD, an entity that has been recognized only relatively recently. To date, seven cases of HIV-NMOSD have been described worldwide, including both AQP4-positive and AQP4-negative patients and the peculiarity of this disease is the concomitant presence in the same patients of hypo- and hyperactivation of the immune system [171]. The mechanism of autoimmunity during HIV infection is unclear; however, autoimmune disease may occur when immune competence is still present around the time of seroconversion or during retroviral therapy. Nevertheless, in one of the reported cases, the onset of NMO was accompanied by a decrease in lymphocyte levels, suggesting the active role of the virus in the development of the disease [171]. It has been observed that HIV can trigger the immune system through the production of alloantigens, the stimulation of immune cells, the induction of interferon gamma production and the release of pro-inflammatory cytokines [171,172]. Moreover, viral products in the lymph nodes and gut mucosal tissue, as well as the presence of HIV DNA in target cells, may activate both the adaptive and the innate immune systems [172,175].

Other pathogenic mechanisms that may be involved are the stimulation of memory T cells by activated HIV T cells [176] and a direct infection by HIV with damage to astrocytes [173].

## 4. MOGAD

The term “MOGAD” encompasses a series of demyelinating diseases of the CNS characterized by optic neuritis, optic neuromyelitis, myelitis, brain/brainstem syndrome or acute disseminating encephalomyelitis (ADEM) together with the presence of anti-MOG IgG antibodies in serum [177,178].

It should be noted that the presence of anti-MOG antibodies can be transient and the disease is sometimes monophasic [179]. Moreover, low antibody titers have a lower predictive value and may indicate a false positive [180].

ADEM is the most common manifestation in the pediatric population and often occurs after infection with EBV, measles, influenza, enterovirus, coronavirus, herpes simplex and, rarely, *Mycoplasma pneumoniae* [181,182,183,184,185,186,187,188,189,190]. Recently, it has been observed that previous infection is more common during the monophasic course of the disease [191].

How microorganisms damage the CNS is still not clear. Both direct and immune-mediated mechanisms have been proposed [190].

A case of MOGAD characterized by longitudinally extensive transverse myelitis occurring three weeks after a streptococcal brain abscess has been described and anti-MOG antibodies were detected only transiently [192]. Similarly to what was observed in patients with MS and in contrast to what was observed in NMOSD patients, higher levels of antibodies against the HERV-W envelope were present in patients with MOGAD [168]. The most probable explanation involves molecular mimicry between epitopes of the HERV-W surface and oligodendrocytes, which is the main cell type implicated in both MS and MOGAD, but not in NMOSD. Indeed, cross-reactivity between MOG and some HERV-W envelope peptides has been reported [193].

A case of severe MOGAD after infection with the Jamestown Canyon virus has been described [194]. This virus can cause fever and meningoencephalitis. In this case, the infection probably triggered an immune response, as has been reported in other cases of MOGAD, especially when the presentation of the disease is ADEM-like in the pediatric population [195].

In Table 3, a selection of studies investigating the role of infectious factors in MOGAD is shown.

## 5. What about SARS-CoV-2?

In recent years, after the beginning of the COVID-19 pandemic, several cases of post-infectious demyelinating diseases have been reported worldwide.

SARS-CoV-2 has direct neurotropism, but its DNA has never been found in the CSF of reported cases; thus, direct damage is not the probable mechanism [196]. On the other hand, the virus can cause an aberrant immune response, increasing the levels of cytokines and chemokines and inhibiting regulatory T cells [197,198]. This mechanism may be related to both cases of relapses and of new-onset demyelinating diseases.

A systematic literature review was conducted to evaluate all demyelinating events described in the context of COVID-19 [196].

The cases described in the literature were as follows: 73 demyelinating events with considerable MS/CIS (10 new diagnoses) with a median time from COVID-19 to the onset of neurological symptoms of 13.5 days; 18 NMOSD events (10 new diagnoses) after a median of 11.5 days from COVID-19 diagnosis; and 28 MOGAD cases (27 new diagnoses) after a median of 6 days from the infection. It should be noted that, in some of the cases, neurological symptoms preceded COVID-19 and the relationship between the SARS-CoV-2 infection and the demyelinating disease should be considered doubtful when more than 6 weeks have elapsed between both events [192]. According to this review, the rates of relapse and onset of MS, NMO and MOGAD associated with SARS-CoV-2 infection are low [196].

## 6. Conclusions

Infectious factors, including viruses, fungi and bacteria, have the potential to act as potent triggers of autoimmunity, playing a significant role in the initiation of demyelinating diseases and their subsequent clinical manifestations.

Currently, it is clear that MS occurs in genetically predisposed individuals when particular environmental factors are present and that previous EBV infection has a determinant role in the pathogenesis of the disease. Indeed, the risk of MS is very low in patients that are EBV seronegative and increases after EBV infection, especially in genetically predisposed individuals. Among the remaining microorganisms that have been associated with the disease, HERV-W has the more important role and it has been considered the missing link between EBV and other environmental factors and the development of MS.

The evidence for the role of microorganisms in the pathogenesis of NMOSD is still not as clear and solid as that available for MS. However, several viruses and bacteria have been associated with the onset or exacerbation of the disease. It is likely that no specific infectious agents are directly responsible for the triggering of NMOSD symptoms. A more probable hypothesis is that any infection that derives in immune system imbalance, causing over-reactivity, production of self-reactive lymphocytes and AQP4, enhanced levels of cytokines and damage to the blood–brain barrier may be involved in the onset or exacerbation of NMOSD.

Little is known about the role of infections in MOGAD, mostly because of the rarity of the disease, its clinical variability and the lack of large study cohorts. Different infections preceding neurological symptoms have been described in case reports and case series, particularly in patients with a monophasic course, none of which have revealed any specific role for the microorganisms.

In conclusion, infectious agents play an important role in the triggering of demyelinating diseases, in particular through an interaction with the immune system. The clearest and best-defined role is that of EBV in the development of MS, which could in fact be considered as one of the rare, severe and long-term complications of EBV infection.

## Figures and Tables

**Table 1:** Selection of studies investigating the role of infectious factors in MS.

MS		
Infectious Agent	Evidence	References
EBV	Definitively established association between EBV infection and MS onset in a wide cohort study	[21]
	High titers of anti-EBNA and VCA antibodies are observed in patients with MS	[25,26,27]
	EBV is a necessary causative agent in the pathogenesis of MS	[28]
	Serum titers of pre-onset anti-EBNA antibodies are strong markers of MS	[29]
	EBNA-1 recognized by MS patient sera induces signs of EAE in a murine model	[30]
	EBNA-1 peptides are cross recognized by anti-MBP antibodies	[31]
	Immunoreactivity against EBV proteins BRRF2 and EBNA-1 is higher in MS; OCBs belonging to MS patients bound both EBV proteins	[32]
	OCBs in CSF belonging to MS patients are able to bind EBNA-1 and EBNA-2 epitopes	[33]
	There is high-affinity molecular mimicry between EBNA-1 and GlialCAM in MS	[34]
	High titers of anti-EBNA increase the risk of MS and are observed between 15 and 20 years before the onset of the disease	[35]
	The risk of MS is notably increased after infectious mononucleosis	[36]
	There is evidence of EBV infection in brain-infiltrating B cells and plasma cells in MS	[37]
	Mutations in EBNA-2 could influence the host response to EBV	[38]
	HLA-DRB1*15:01 acts as coreceptor for EBV infection of B cells	[39]
	Specific EBNA-1 antibodies and HLA-DRB1*1501 interact in the MS risk	[40]
	EBNA-3 blocks the activation of vitamin D receptor-dependent genes	[41]
	EBNA-1 antibodies correlate with radiological disease activity	[42,43]
	The cellular immune response to EBV decreases during ocrelizumab treatment	[44]
	Teriflunomide inhibits cellular proliferation in EBV-transformed B cells	[45]
HERV	The envelope protein of HERV-W has been detected in serum, brain, perivascular infiltrates and macrophages of patients with MS	[46,47,48]
	HERV mRNA has been found in the brain lesions, CSF and blood cells of individuals with MS	[49,50]
	The expression of HERV is increased in patients with active MS	[51,52]
	There is evidence of molecular mimicry between HERV-W envelope protein and myelin proteins	[53]
	HERV may activate the host immune response by acting as an agonist of human toll-like receptor 4	[54]
	The HERV-H envelope and gag proteins have been reported to be present in the serum of MS patients	[55]
	HERV-W could act as effector in MS pathogenesis through its activation during EBV infection	[56]
	EBV transactivates the HERV-K18 that encodes a superantigen	[57]
	HERV-W DNA copy number was found to be higher in MS patients and was inversely correlated with vitamin D level	[58]
	Interferon beta may decrease the expression of HERV-W	[59]
	Natalizumab inhibits the expression of HERV-W	[60]
	A new drug tested in a phase II clinical trial for MS, known as GNbAC1, is able to block the HERV-W-dependent inflammatory cascade	[61,62]
HHV-6	OCBs specific against HHV-6 have been identified in patients with MS	[63]
	Pro-inflammatory cytokines are higher in HHV-6 infected patients and HHV-6 positivity is associated with higher disability	[64]
	Anti-HHV-6 IgG titers significantly predict subsequent relapse risk in MS	[65]
	The lymphoproliferative response to HHV-6A is increased in MS	[66]
	There is an increased prevalence of HHV-6A in MS	[67]
	MBP cross-reacts with HHV-6 antigens; thus, there is evidence of a molecular mimicry	[68]
	Increased serological response against HHV-6A is associated with the risk of MS	[69]
	There is an interaction between environmental factors and high titers of anti-HHV-6A antibodies in the risk of MS	[70]
	HHV-6A is a risk factor for MS	[71]
Gut Microbiota	Gut bacteria from patients with MS have pro-inflammatory properties	[72]
	Disease-modifying therapies alter gut microbial composition in MS	[73]
	Interferon beta can cause an increase in *Prevotella*	[74]
	Gut microbiota differs from MS and controls. *Enterobacteriaceae* and several *Clostridium* species are associated with progressive course and disability	[75]
	There is an alteration of gut microbiota in MS patients, with an over-representation of *Saccharomyces* and *Aspergillus*	[76]
Fungi	First evidence of fungal infection in CNS tissue of MS patients, with detection of fungal DNA	[77]
	The specific enzyme activity of *Candida albicans* is greater in MS patients and correlates with disease severity	[78]
	Fungal antigens and antibodies against several *Candida* species have been detected in CSF of MS patients	[79]
	Calprotectin levels in the CSF reflect disease activity	[80]
	Some improvement in MS symptoms was observed in MS patients after treatment with antifungal drugs	[81]
MAP	MAP peptides are cross-recognized by anti-MBP antibodies	[31]
	MAP is associated with MS in Sardinian population	[82,83]
	MAP is associated with MS in Japanese population	[84]
	Epitopes of MAP2694 homologous to TCR are highly recognized in MS	[85]
	Human IRF 5 homologous epitopes of MAP induce a specific immune response	[86]
	There is no association between the haplotypes predisposing to MS and MAP positivity	[87]
CMV	CMV can intensify the symptoms in MS patients	[88]
	CMV seropositivity is negatively associated with MS	[89,90]

MS: multiple sclerosis; EBV: Epstein–Barr virus; EBNA: Epstein–Barr nuclear antigen; VCA: viral capsid antigen; EAE: experimental autoimmune encephalomyelitis; OCBs: oligoclonal bands; MBP: myelin basic protein; CSF: cerebrospinal fluid; HERV: human endogenous retrovirus; HHV: herpes human virus; CNS: central nervous system; MAP: mycobacterium avium paratuberculosis; IRF: interferon regulatory factor; CMV: cytomegalovirus.

**Table 2:** Selection of studies investigating the role of infectious factors in NMOSD.

NMOSD		
Infectious Agent	Evidence	References
TB	Case report: a patient with NMOSD experienced relapses following episodes of pulmonary TB	[155]
	There is no association between NMO and pulmonary TB in a Chinese cohort	[156]
	There is an association between NMO and pulmonary TB in a South-African cohort	[157]
	Two cases of NMOSD with onset temporally related to pulmonary TB	[158]
	Antituberculosis treatment reduced relapses in patients with steroid-refractory NMO and led to neurological recovery	[159]
	Pulmonary TB was associated with NMOSD in 5 out of 24 Brazilian patients	[160]
*H. pylori*	High titers of anti *H. pylori* antibodies are observed only in AQP4-positive patients	[161]
	High titers of anti *H. pylori* antibodies are observed in both AQP4-positive and -negative patients, with stronger association with the first group	[162]
	A higher prevalence of *H. pylori* infection was described in AQP4-positive compared to AQP4-negative patients	[163]
	There is no difference between AQP4-negative patients and healthy controls in terms of the frequency of *H. pylori* infection	[164]
EBV	Anti-EA IgG are higher in NMO patients than in MS patients and healthy controls, both in sera and CSF	[165]
	NMO is associated with the reactivation of EBV	[166]
HERV	Patients with NMOSD have lower levels of anti-HERV antibodies compared to patients with MS and to healthy individuals	[167,168]
Gut microbiota	*Clostridium perfringens* is abundant in individuals with NMO	[169]
	There is a potential molecular mimicry between *Clostridium perfrigens* and AQP4	[170]
Fungi	High in vitro immune reactivity to *Escherichia coli* is correlated with disability in NMO	[148]
HIV	Characterization of six cases of patients with HIV developing NMOSD	[171]
	HIV is able to activate several cellular lines of the immune system	[172]
	Characterization of two cases of patients with HIV developing NMO	[173]

NMOSD: neuromyelitis optica spectrum disorder; TB: tuberculosis; EBV: Epstein–Barr virus; EA: early antigen; HERV: human endogenous retrovirus; HIV: human immunodeficiency virus.

**Table 3:** Selection of studies investigating the role of infectious factors in MOGAD.

MOGAD		
Infectious Agent	Evidence	References
HERV	Higher levels of antibodies against HER-W were present in MOGAD than in NMO	[168]
	There is a cross-reactivity between MOG and HERV-W proteins	[193]
Measles	Case report: ADEM associated with measles	[183]
Influenza	Case report: ADEM associated with influenza A H1N1 infection	[184]
Coronavirus	Case report: ADEM with detection of coronavirus in CNS	[186]
Enterovirus	Case report: ADEM with positive PCR for enterovirus in the CSF	[185]
*Mycoplasma pneumoniae*	Description of cases reporting MOGAD following *Mycoplasma pneumoniae* infection	[189,190]
Jamestown Canyon virus	Case report: MOGAD with bilateral corticospinal tract lesions following infection with Jamestown Canyon virus	[194]
Streptococcus	Case report: MOGAD transverse myelitis following brain abscess	[192]
EBV	Case report: anti-MOG positive ADEM following infectious mononucleosis	[187]
Herpes simplex	Case report: MOGAD (bilateral optic neuritis and meningoganglionitis) following a genital herpes simplex virus infection	[188]

MOGAD: Myelin oligodendrocyte glycoprotein antibody disease; HERV: human endogenous retrovirus; NMO: neuromyelitis optica; ADEM: acute disseminating encephalomyelitis; CNS: central nervous system; CSF: cerebrospinal fluid; EBV: Epstein–Barr virus.

## Data Availability

Not applicable.
